# Dearth of polymorphism associated with a sustained response to selection for flowering time in maize

**DOI:** 10.1186/s12862-015-0382-5

**Published:** 2015-06-07

**Authors:** Eleonore Durand, Maud I Tenaillon, Xavier Raffoux, Stéphanie Thépot, Matthieu Falque, Philippe Jamin, Aurélie Bourgais, Adrienne Ressayre, Christine Dillmann

**Affiliations:** INRA, Ferme du Moulon, Gif sur Yvette, 91190 France; Univ Paris-Sud, Ferme du Moulon, Gif sur Yvette, 91190 France; CNRS, Ferme du Moulon, Gif sur Yvette, 91190 France

**Keywords:** Quantitative genetics, Selection response, Heritability, Standing variation, Epistasis

## Abstract

**Background:**

Long term selection experiments bring unique insights on the genetic architecture of quantitative traits and their evolvability. Indeed, they are utilized to (i) monitor changes in allele frequencies and assess the effects of genomic regions involved traits determinism; (ii) evaluate the role of standing variation versus new mutations during adaptation; (iii) investigate the contribution of non allelic interactions. Here we describe genetic and phenotypic evolution of two independent Divergent Selection Experiments (DSEs) for flowering time conducted during 16 years from two early maize inbred lines.

**Results:**

Our experimental design uses selfing as the mating system and small population sizes, so that two independent families evolved within each population, Late and Early. Observed patterns are strikingly similar between the two DSEs. We observed a significant response to selection in both directions during the first 7 generations of selection. Within Early families, the response is linear through 16 generations, consistent with the maintenance of genetic variance. Within Late families and despite maintenance of significant genetic variation across 17 generations, the response to selection reached a plateau after 7 generations. This plateau is likely caused by physiological limits. Residual heterozygosity in the initial inbreds can partly explain the observed responses as evidenced by 42 markers derived from both Methyl-Sensitive Amplification- and Amplified Fragment Length- Polymorphisms. Among the 42, a subset of 13 markers most of which are in high linkage disequilibrium, display a strong association with flowering time variation. Their fast fixation throughout DSEs’ pedigrees results in strong genetic differentiation between populations and families.

**Conclusions:**

Our results reveal a paradox between the sustainability of the response to selection and the associated dearth of polymorphisms. Among other hypotheses, we discuss the maintenance of heritable variation by few mutations with strong epistatic interactions whose effects are modified by continuous changes of the genetic background through time.

**Electronic supplementary material:**

The online version of this article (doi:10.1186/s12862-015-0382-5) contains supplementary material, which is available to authorized users.

## Background

Evolutionary pressures, including selection, determine the level of genetic and epigenetic variation within populations. This variation, in interaction with the environment, results in continuous phenotypic variation for life-history traits. For a given trait, the response to selection is measured by the deviation of phenotypic mean from one generation to another. It depends on the trait heritability [1-3] measured as the additive variance that is transmitted from parents to offspring [4] divided by the total phenotypic variance. On the one hand, trait heritability depends on the susceptibility of the trait to environmental fluctuations. On the other hand, it depends on the total genetic and epigenetic variation as well as the modalities of transmission of those components. For example, interactions between alleles, that are not transmitted in panmictic populations, and epigenetic variants, that are non-mendelian inherited, contribute to the total phenotypic variation but may result in decreased heritability [5].

Genetic or epigenetic variance comprises additive, dominance and epistatic variance components. The two latter variance components imply interactions between alleles. Additive variance instead designates the effect of an allele as measured by its average deviation from the population phenotypic mean. A long-standing controversy revolves around the relative importance of additive versus non-additive (dominance and epistatic) effects. Its last avatar, the so-called "missing heritability" paradox, arose from genome-wide linkage and association mapping studies. Indeed, for a broad range of traits and organisms, locus-by-locus studies have revealed that genomic polymorphisms associated with phenotypic variation of complex traits typically account for a small fraction of additive variance [6] while additive variance is known to be by far the greatest contributor of the total genetic variance [5, 7, 8].

Multiple non-exclusive hypotheses have been proposed to resolve this paradox among which an excess of rare alleles limiting detection power [9], and a prominence of undetectable epistatic interactions that could significantly contribute to phenotypic variation [6]. Growing knowledge on biological pathways and gene networks indeed points to pervasive gene interactions at the molecular levels (for a review, [10, 11]). Such interactions appear as key features of traits architecture and quantitative variation ([12]). However the amount of additive genetic variance in a population tightly depends on allele frequencies. Typically, when allele frequencies are distributed towards extreme values, as predicted by the the neutral mutation model [13], most of the genetic variance within population is expected to be additive regardless of existing dominance and epistatic interactions [8]. In other words rare variants affect less the additive variance than the non-additive variance.

Sustainability of the response to selection depends on how the additive variance changes through time. It is affected by multiple factors, the number, the frequency, and the effect of each allele influencing the trait [14] together with the interactions introduced by epistasis [15], the correlations induced by linkage disequilibrium [16, 17], the strength of selection, the rate of occurrence and distribution of new mutations [18, 19], and the strength of random genetic drift [20, 21]. Unfortunately because of allele segregation at meiosis, changes in mean and variance components can not be resolved analytically even in the simplest case of one population submitted to directional selection on a single trait [18, 22]. Additional assumptions are needed and various models have been proposed to predict long-term changes. The infinitesimal model - in which traits are governed by an infinite number of independent loci with small effects - predicts changes in trait value with negligible changes in allele frequencies and genetic variance [23]. In contrast, whenever the population size or the number of loci is finite, predictions include a decrease of genetic variance and of the response to selection until a steady state is reached, i.e. selection/mutation/drift equilibrium [20]. Moreover, non-additive interactions generate long term measurable additive variance by small changes in allele frequencies and their underlying interactions, and corresponding changes in the response rate [15, 24-26]. Finally, genetic background changes in the presence of genotype by environment interactions [27] may result in canalization, i.e. decreased variance around phenotypic optimum [28]. Hence, one important feature of the dynamics of the response to selection is that non-additive interactions may have a significant impact in the long-term [29].

Divergent selection experiments on complex traits have contributed to dissect the determinants of the response to selection [30-34]. In plants, one of the best example is the Illinois maize long-term selection experiment that has been conducted over 140 generations. Initiated in 1896, this experiment aimed at selecting an open-pollinated variety for kernel protein and oil content in two directions, low and high value of each trait. The observed response to selection has been steady over time indicating no exhaustion of variability [35]. In flour beetles, continuous selection over 130 generations on pupa weight, starting from a heterogeneous background, has also resulted in constant increase of phenotypic value with a small decline in genetic variance. The maintenance of variability was best explained by the input of new mutations [36], or perhaps new epimutations. In canola, the input of new variation have been measured by artificially selecting respiration intensity from a doubled haploid background for 5 generations [37]. The results revealed significant changes in respiration intensity pointing to the role of *de novo* epimutations. Altogether these examples reinforce the idea that existing or *de novo* variability are not limiting factors when selecting for complex traits in eukaryotes.

But what are the mechanisms underlying the response to selection? Access to genomic information offers exciting prospects to answer this question and unravel the genetic bases of phenotypic differentiation. So far, only few studies have assessed genome wide effects of long-term divergent selection in higher organisms. The first one, by [38] reports the results of artificial selection for accelerated development on outbred populations of *Drosophila* with population sizes of about 1500 individuals and effective population sizes around 230 [39]. It found no evidence for fixation of unconditionally advantageous alleles after 600 generations. Among other explanations, [38] propose that fluctuations of selective coefficients over time may explain the observed pattern. In other words, selection would proceed via multiple alleles of small effects each contributing transiently to the fitness increase [40]. Similar results were obtained from the analysis of the Golden Glow maize long-term selection population [41]: 30 generations of selection for increased number of ears per plant with an effective population size around 500 led to a >3-fold increase without allelic fixations but multiple instances of soft sweeps. In contrast, the maize Krug Yellow Dent long-term divergent seed-size selection experiment resulted in a combination of soft and hard sweeps associated with the fixation of alleles of opposite effects clustered in two genomic regions [42]. This experiment was conducted with similar demographic size as [41] but slightly lower effective population size (<300). Interestingly, genomic regions that responded to selection did not overlap with QTLs for seed weight detected in association studies. Genome-wide study of allele frequency changes have also been performed in the Virginia chicken lines experiment in which selection was applied bi-directionally on body weight for 50 generations [43] with an effective population size around 35. This study revealed dramatic effects of selection along the genome with multiple instances of complete sweeps whereby alternative alleles were fixed within the high- and low-weight population [44]. The reasons behind the differences observed between studies are unclear but may in part be explained by differences in effective population sizes with complete sweeps associated with smaller populations.

In this manuscript we describe phenotypic evolution of two independent Divergent Selection Experiments (DSEs) conducted during 17 generations from two maize inbred lines and investigate associated genetic changes in the first seven generations using genome-wide assessment of polymorphisms using both Methyl-Sensitive Amplification Polymorphisms (M-SAP) and Amplified Fragment Length Polymorphisms (AFLP). The target trait of selection is flowering time. It is therefore fitness-related and encoded by numerous loci with around 80 Quantitative Trait Loci (QTLs) detected for female and male flowering [45]. The seven first generations of selection were described in a previous paper [46]. While the observed response is sustainable through time in all populations, we found only dozens of segregating polymorphisms in those initially nearly-fixed genetic backgrounds. All were present at the start of the experiments as residual heterozygosity. Linkage and association mapping within our experimental setting reveal significant effects of tightly-linked markers. We discuss our findings from a somehow provocative perspective of missing polymorphisms that parallels previous debates on missing heritability.

## Results

### Description of the DSEs’ experiment

**Fig. 1:**
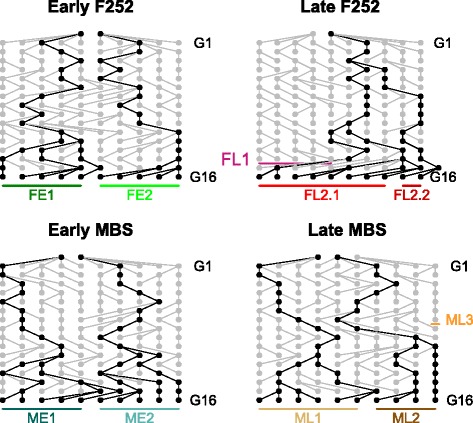
Pedigree of the progenitors in the *F*252 and *MBS* DSEs. Each dot represents one progenitor, from generation G0 to generation G16. Pedigrees of generation *G*16 are shown in black. At each generation, 100 selfing progenies from each progenitor were evaluated, and between 0 to 3 were selected to constitute the progenitors of the next generation. Note that 11 progenitors were selected at generation *G*15 in the Late F252. At generation *G*16, each DSE encompasses two Early and two Late families that were derived independently since generation *G*0, except for the Late F252 population, for which the two families were derived since generation *G*4

We conducted 2 independent DSEs for flowering time from 2 commercial maize inbred lines, *F252* and *MBS847* (*MBS*). As a result we obtained for each DSE two derived populations of Early- and Late-flowering progenitors (plants) previously identified as Early *F252*, Late *F252*, Early *MBS*, Late *MBS* [46]. Within each DSE, selected plants were selfed at each generation. We traced back the *F252* and *MBS* pedigrees from generation 16 (*G*16) to the start of the DSEs (*G*0) (Fig. 1) with a special emphasis on generation *G*6 to compare with our previous results published in [46]. At generation *G*16, pedigrees of Late and Early populations each encompassed two families, named respectively FE1, FE2, FL2.1, FL2.2 for Early and Late *F252*, and ME1, ME2, ML1, and ML2 for Early and Late *MBS*. The families ME1, ME2, ML1 and ML2 are independent and derived from 4 different ancestors at generation *G*0 chosen within the initial *MBS* seed lot. Note that at generation *G*7, the Late *MBS* population also encompassed a third independent family named ML3, that derived from another independent ancestor at generation *G*0. Within the *F*252 DSE, there was 4 independent families at generation *G*7 named FE1, FE2, FL1 and FL2, derived from 4 different ancestors at generation *G*0 chosen within the initial *F*252 seed lot. FL1 became extinct at generation *G*14 (Fig. 1). Generation *G*16 therefore encompasses 2 subfamilies FL2.1 and FL2.2 derived from two lineages of FL2 that splitted from a single progenitor selected at generation *G*3. Altogether, the two DSEs are represented by 10 families (FE1, FE2, FL1, FL2.1, FL2.2, ME1, ME2, ML1, ML2, ML3) evolving throughout the pedigrees.

### Selection responses over sixteen generations

At each generation, progenitor flowering dates were recorded as the 12 earliest or latest plants in their progeny for Early and Late progenitors respectively. We used these records to investigate the response to selection treating each family independently. The response was corrected by the year effect using yearly phenotypic records from the parental inbred lines as control. Comparisons between DSEs were achieved by centering around the average value at generation *G*1 in *F*252 and *MBS* populations, and standardizing by the corresponding residual deviation of the control.

**Fig. 2:**
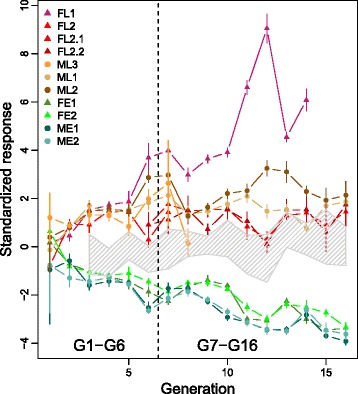
Response to selection in the *F*252 and *MBS* DSEs. The average flowering time of each family as estimated by difference to the first generation per unit of control residual standard deviation is plotted against time in generation. A separate color code for lines and points has been attributed to each family. Vertical lines indicate standard deviation within families. Data from the *F*252 and *MBS* DSEs have been treated separately, except for control lines. The shaded area represents the range of flowering time variation in control lines (data available only from generation G3)

We observed a significant and sustained response to selection during the first 7 generations (*G*0 to *G*6, Fig. 2) in all families with an average gain comprised between 3.66 and 16.04 and an average loss comprised between -6.04 and -11.22 degree days per generation (Table 1). In the Early populations, response was similar in both genetic background, *F*252 or *MBS* (Table 1). In the Late populations, all *MBS* and *F*252-derived families exhibited a similar pattern (Fig. 1) with a greater response to selection compared to Early populations and less variation among generations (elevated *R*^2^ values, Table 1) for ML2 et FL1. FL1 was particularly noteworthy. It exhibited the greatest increase in the response to selection (+16.04, Table 1) and corresponded to the previously described Late-VL *F252* (VL =Very Late) while FL2 corresponded to Late-NVL *F252* (NVL =Non Very Late) [46].

**Table 1:** Response to selection and variance components between generations G0 and G6

Family	df	Response	stde	p-value	*R* ^2^	Median(*H* ^2^)	Max(*H* ^2^)
FL1	330	16.04	1.20	0.00000	0.35	0.32	0.64
FL2	310	3.66	1.10	0.00104	0.03	0.01	0.30
ML1	226	8.89	1.51	0.00000	0.13	0.00	0.28
ML2	303	12.38	1.19	0.00000	0.26	0.17	0.44
ML3	171	7.68	2.05	0.00025	0.07	0.05	0.29
FE1	316	-8.47	0.82	0.00000	0.25	0.02	0.35
FE2	313	-6.04	0.80	0.00000	0.15	0.13	0.19
ME1	332	-11.22	1.14	0.00000	0.22	0.07	0.25
ME2	326	-10.32	1.17	0.00000	0.19	0.02	0.40
F-CONTROL	142	-0.39	1.49	0.80	0.00		
M-CONTROL	137	-2.68	2.21	0.23	0.00		

The response to selection was analyzed separately in each family from progenitor phenotypic data after correction for block and year effects. Response (degree-days per generation) is measured as the slope of the linear regression of flowering date (thermal time) on generations of selection (see Methods section). *df* is the number of degrees of freedom. Note that variation between families mainly denotes variations in the number of progenitors. *stde* is the standard error for the response, and *R* ^2^ the adjusted R-squared of the regression model. Broad-sense heritability (*H* ^2^) was estimated at each generation as the ratio of the variance between progenitors belonging to the same family on the total phenotypic variance

The pattern observed from *G*7 through *G*16 was different. Early populations continued to respond to selection with an average loss comprised between -4.36 and -5.78 degree-days per generation (Table 2) that was similar accross genetic backgrounds, but lower than from *G*0 to *G*6 (Table 1). Late populations instead ceased to respond to selection except for FL1 (+13.02 degree days per generation) and ML2 (+2.65). Progenitors from the FL1 family were discarded from the experiment at G14 because the plants flowered too late in the season to produce viable offspring. In all other Late families, the variation between generations was much higher than the average response (non significant *R*^2^, Table 1). As exemplified by the FL2.1 and FL2.2 families, the response varied across generations alternating positive and negative slopes (Fig. 2).

**Table 2:** Response to selection and variance components between generations G7 and G16

Family	df	Response	stde	p-value	*R* ^2^	Median(*H* ^2^)	Max(*H* ^2^)
FL1	427	13.02	1.05	0.00000	0.26	0.08	0.61
FL2.1	345	0.12	0.57	0.83	0.00	0.21	0.37
FL2.2	196	0.51	0.87	0.56	0.00	0.10	0.56
ML1	461	-0.20	0.44	0.65	0.00	0.03	0.52
ML2	471	2.65	0.62	0.00002	0.04	0.38	0.70
FE1	593	-4.36	0.26	0.00000	0.33	0.21	0.33
FE2	480	-4.68	0.27	0.00000	0.37	0.16	0.68
ME1	580	-5.78	0.28	0.00000	0.43	0.30	0.55
ME2	565	-4.71	0.31	0.00000	0.29	0.24	0.53
F-CONTROL	195	-0.48	0.74	0.519	0.00		
M-CONTROL	211	1.54	0.60	0.0111	0.03		

Abbreviations are the same as Table 1

Altogether, the response to selection was asymmetrical. In the Early populations, the response was weaker but almost linear throughout the experiment. In contrast in Late populations, the response was strong at the beginning and almost ceased after G6 except for FL1 and ML2. To test whether the lack of response to selection for lateness were due to the exhaustion of genetic variability within families, we measured the genetic variation between progenitors of the same family over generations. Statistics for the so-called within family broad-sense heritability (*H*^2^) are given in Table 1 and Table 2. There were on average 5 different progenitors within each family each year. A value of *H*^2^ significantly different from zero indicates significant differences between progenitor genotypic means with regard to the residual variation between plants from the same progenitor. Although heritability medians were relatively low, ranging between *median*(*H*^2^)=0.00 and *median*(*H*^2^)=0.38, heritabilities significantly differed from zero in all families in at least one generation (*max*(*H*^2^)>0, Table 1 and 2). This corresponded to bursts of genetic variation resulting in a *L*-shaped distribution of *H*^2^ across generations and families (see Additional file 1: Figures S3 and S4) and high values for the maximal *H*^2^ within families, ranging from 0.19 to 0.70. It is remarkable that there was a tendency for the median of *H*^2^ to be higher between generations *G*7 and *G*16 than between generations *G*1 and *G*6 (Table 1 and Table 2). Note that transcient increase of *H*^2^ is expected from fixation of initial polymorphisms in selfing populations. Starting from an heterozygote, the variance between progenitors is expected to increase, and the variance within progenitor is expected to decrease due to selfing [47]. Overall, our observations indicated no significant decrease in genetic variance neither within Early nor within Late families. Hence, decrease in genetic variance cannot account for the lack of response to selection between *G*7 and *G*16 in the FL2.1, FL2.2, ML1, ML2 families. At generations *G*7 and *G*17, we performed flowering time measurements for all plants and compared them with the average flowering time of progenitors selected to form *G*8 and *G*18 (data not shown). It confirmed that there was significant variation both between and within progenitors, even within the Late families. However, selection was inefficient because the most extreme plants i.e. the latest, did not contribute to *G*8 and *G*18. This was due to their reduced fertility in our environmental conditions.

Altogether, we observed a significant response to selection in both directions during the first 7 generations of selection. In the Early families, the response was linear through 17 generations of selection and sustained by a constant input of additive genetic variance within families. In contrast, selection has likely reached physiological limits in Late families after generation *G*7 since we verified the maintenance of genetic variation within each family.

### Markers discriminate early and late families

In order to assess the genetic and epigenetic determinants of the response to selection, we combined both Methyl-Sensitive Amplification Polymorphisms (M-SAPs) and Amplified Fragment Length Polymorphisms (AFLPs) to genotype all progenitors selected during the first 7 generations. Both techniques are commonly used for genome-wide polymorphism detection because they are extremely cost-effective, they generate a large amount of markers, and display a strong discrimination power at low taxonomic level such as strain/inbred material [48]. The main drawback of AFLPs is to be dominant markers. However in our case, genealogical information facilitates genotype calling. We developed a likelihood framework to infer genotypes from AFLP phenotypes and explicitely accounted for pedigree information and various sources of experimental errors (Additional file 2).

**Fig. 3:**
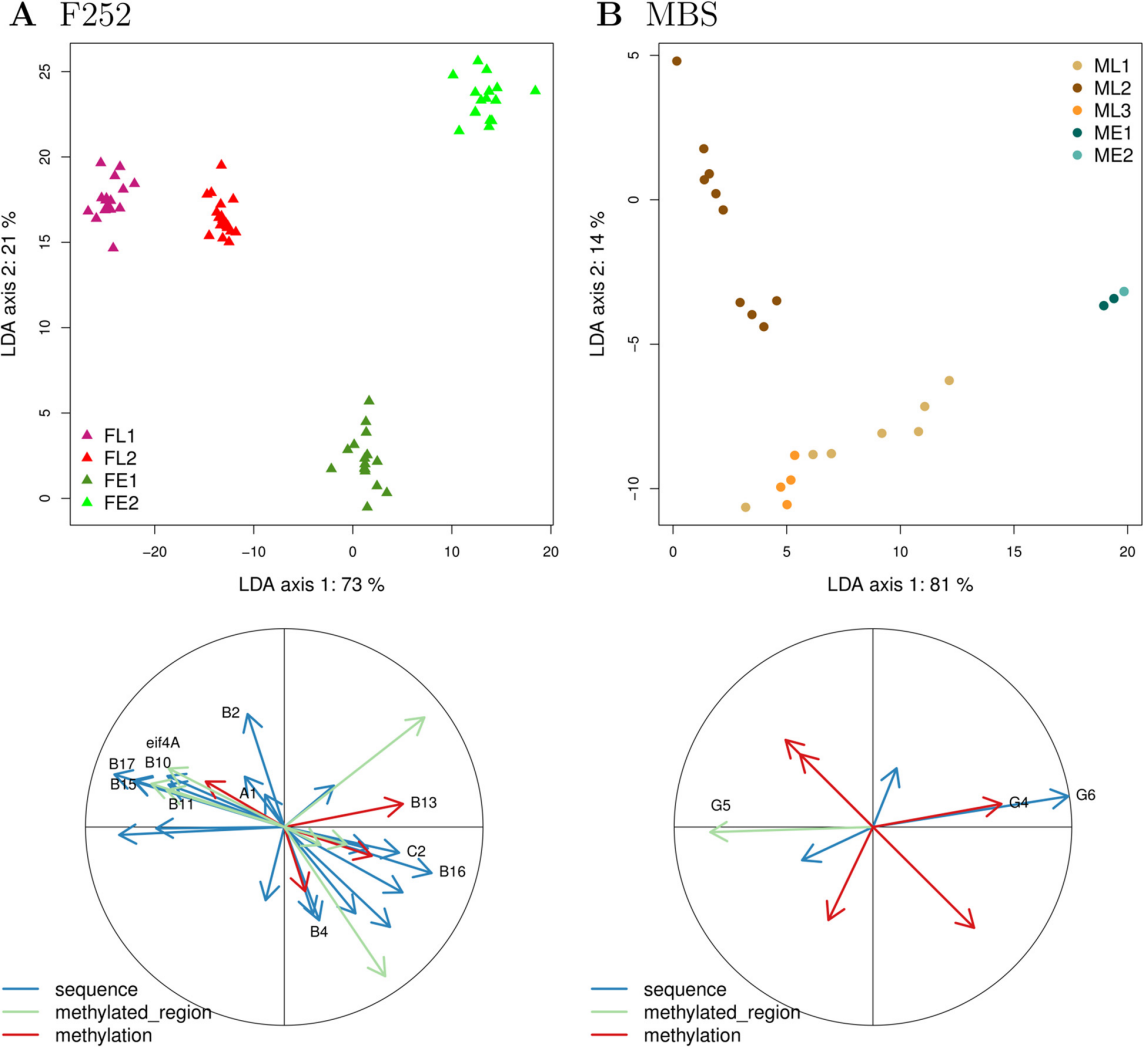
Differenciation among families as revealed by markers during the 7 first generations of selection. Linear Discriminant Analysis using families as discriminant factor. Progenitors (upper panels) and markers’ contribution (lower panels) are represented in **a** F252 DSE. **b** MBS DSE for the first two discriminant axes. Each datapoint represents a single progenitor. For the markers, the type of polymorphism (sequence, methylation, or sequence polymorphism within a methylated region) is indicated. Only markers significantly associated with phenotypic variation are named

Given the highly inbred material used to start our DSEs, we expected a restricted number of markers. In maize inbred lines, the nucleotide residual heterozygosity is classically estimated at few percents [49] with variations along the genome [50]. In our material, the sustainability of the response to selection clearly indicated a substantial amount of segregating polymorphisms either from standing genetic variation or from *de novo* mutations that occurred during the course of the experiment [46]. Using 11 and 9 primer combinations for *F252* and *MBS* DSEs respectively, we identified 33 markers segregating in *F252*, and only 9 in *MBS* (Fig. 3a and b). Within the *F*252 DSE, 4 polymorphisms were methylation-based polymorphisms while 29 were either sequence-based or located in a methylated region (Fig. 3a). Within the *MBS* DSE, 5 were methylation-based polymorphisms (Fig. 3b). All markers were present in the initial seed lots as residual heterozygosity. Some markers, 13 out of 33 and 1 out of 9, segregated both in Early and Late populations respectively, while the remaining (20 out of 33 and 8 out of 9) only segregated within one population. In all families, the initial heterozygosity decreased at a rate that was not different from (1/2). As a result, all progenitors were homozygous for one or the other allele at each locus at generation G6. The dataset was complemented with information from the previously obtained PCR marker at locus eIF-4A associated with variation of flowering time [51].

While scarce, markers offered a surprisingly high discrimination power. Discriminant analyses indeed revealed that 94 % to 95 % of the variation between families was explained by the two first axes with different types of markers equally contributing to their definition (Fig. 3). In *F252*, the first axis discriminated all 4 families, the Early FE1, FE2 from the Late FL1, FL2 while the second axis essentially distinguished the 2 Early families. In *MBS*, the first axis discriminated the Early from the Late population while the second axis discriminated the Late families, the Early families being indistinguishable. Those results were confirmed by an analysis of molecular variance on allele frequencies [52]. In *F*252, 41 % (p-value=0.0002) of the variance was observed between Late and Early populations, and 23 % (p-value=0.0004) between families within populations. In *MBS*, 57 % (p-value =0.0002) of the variance was between populations and 16 % (p-value =0.01) between families within populations. Therefore variance among families within populations was consistently smaller than variance among populations. Such pattern is unexpected under a pure drift model.

To summarize, all the markers found were present as residual heterozygosity in the initial inbred lines. They corresponded to sequence as well as to methylation polymorphisms and discriminated Late from Early populations. In other words, independently derived families within populations more often fixed the same alleles than families from different populations. Our results therefore advocate for a dominant role of selection over drift despite small effective population sizes.

### Association and Linkage mapping reveal a dearth of causal polymophisms

**Fig. 4:**
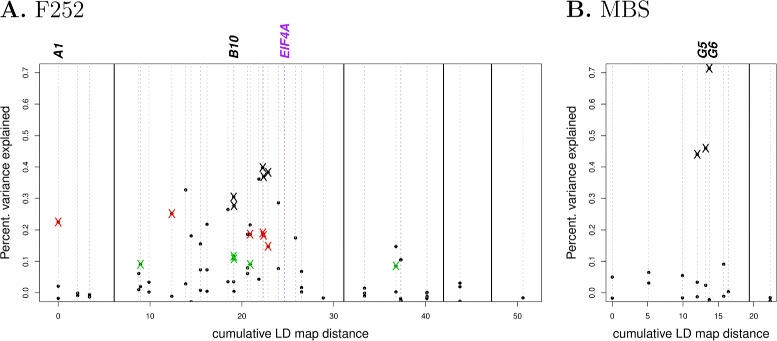
Marker-based Linkage Disequilibrium map and percentage of flowering time variations explained by each marker. Markers are ranked according to their position along the LD map (see text). Vertical lines separate linkage groups. *X* indicate a significant effect either between Late and Early populations (Black), or within Early (Green) or Late (Red) populations. **a** F252 divergent selection experiment. **b** MBS divergent selection experiment. Names of the four markers used in Fig. 5 are indicated on top

We calculated the linkage disequilibrium between the markers associated with flowering time with the purpose of clustering and ordering them in linkage groups. We found 5 distinct linkage groups for *F*252 and two for *MBS* (Fig. 4 a and b). Among the 5 *F*252 linkage groups, one contained 17 markers clustered in a region bearing the gene *eIF*-4A associated with flowering time variation both within the *F252* pedigrees and a large panel of maize inbred lines [51].

**Fig. 5:**
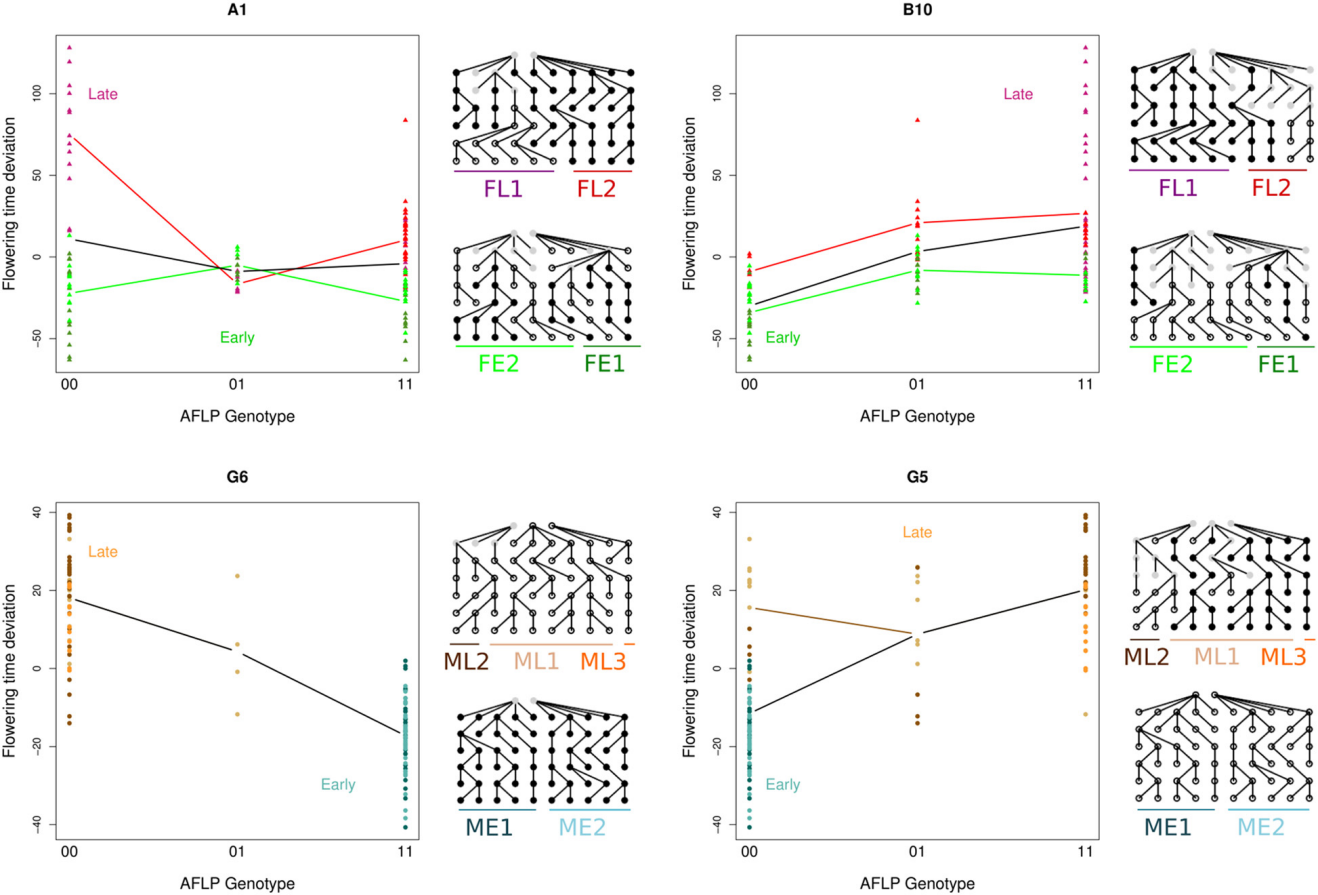
Examples of polymorphisms’ segregation and association with flowering time. Results are presented for markers A1 and B10 from the F252 DSE (top panels), and markers G5 and G6 from the MBS DSE (bottom panels). **Left of each panel**: relationship between flowering time deviation and genotype at the marker. Each datapoint represents a single progenitor. Colors codes used for families are the same as in Fig. 1. Lines connect population means. Black lines connect overall means. **Right of each panel**: corresponding pedigrees of the DSE progenitors and genotypes. Heterozygotes are in grey and homozygotes for the presence of the band are in black

For each marker, we measured its additive and dominant effect using flowering data gathered along pedigrees. We assessed its significance using gene dropping simulations, thereby explicitly accounting for pedigree information. In *F252* populations, among 33 markers, 10 exhibited a significant additive effect while none displayed a dominant effect. Among the 10 significant markers, 5 discriminated Late versus Early populations, but also Early or Late families. Altogether, 4 markers had a significant effect within Early populations, 5 had a significant effect within Late populations, and one was significant both within Early and within Late populations (Fig. 4a). The magnitude of effects changes between the Late and the Early populations. It was below 5 degree-days within Early but above 15 within Late (data not shown). Consistently, the percentage of variance explained by the markers was greater within Late (0.15<*R*^2^<0.25) than within Early (0.02<*R*^2^<0.12) populations. This observation was consistent with a greater response within the Late families ML2 and FL1 compared with the Early during the 7 first generations of selection (Table 1). Note that most significant associations (8 out of 10) corresponded to markers from the same linkage group which also contains the *eIF*-4A gene. In *MBS* populations, among 9 markers, a subset of 3 exhibited a significant additive effect (Fig. 4b) with a range of magnitude comparable to what we reported for *F252* from 5 degree days up to 18 degree days (data not shown). Note the elevated *R*^2^ value observed for marker *G*6.

To illustrate the results, some examples of the segregation of alleles and association with flowering time are shown for 2 markers from *F*252 and *MBS* respectively (Fig. 5). Markers A1 and B10 were polymorphic within both Early and Late *F*252 populations. For marker A1, the loss of the band in the *FL*1 family was associated with very late flowering time, while it had no apparent effect in the Early families. For marker B10, the presence of the band was associated with lateness in both Early and Late families. Consistently, the band had been lost during the selection process in most Early progenitors. In the *MBS* DSE, the presence of the band at marker G6 was strongly associated with earliness. Except for generations *G*0 and *G*1, this marker perfectly discriminated the Early (band present) and Late (band absent) families. Marker G5 exhibited a similar pattern but the presence of the band was associated with lateness. The band had been lost as soon as generation *G*0 in the Early families while it still segregated in the Late families.

When computed on the subset of markers that were found significantly associated with flowering time variation, the analysis of molecular variance revealed an increase of the between-population variance and a corresponding decrease of the within-family residual variance. In *F*252 DSE, 55 % (p-value = 0.0004) of the variance was observed between Late and Early populations, and 21 % (p-value = 0.005) between families within populations. In *MBS*, 78 % (p-value = 0.0002) of the variance was observed between populations and 14 % (p-value =0.0004) between families within populations. Hence, marker segregation matched the direction of selection, the loss of polymorphism being faster within families for markers associated with flowering time variation.

Altogether, our results demonstrated that about one third of the markers were associated with differences in flowering time. Despite the fact that the families were derived independently, marker segregation depended on the direction of selection, and ultimately discriminated Late from Early populations irrespective of the family. Within *F*252 DSE, all markers associated with flowering time variation but two exhibited tight linkage with the *eIF*-4A gene.

## Discussion

Our ongoing Divergent Selection Experiments (DSEs) from two maize inbred lines with flowering time as target trait have now been conducted for 18 years. We presented here the results obtained from generations G0 to G16. Altogether, we observed a significant response to selection in both directions that is comparable for the two DSEs and similar to the ones classically observed for DSEs in several respects. First, the phenotypic mean was driven several times the standard residual deviation away from its starting value (Fig. 2). Second, response to selection was asymmetrical and depended on the direction of selection. Third, we observed fast fixation of initial polymorphisms. Large response to selection is a common outcome of selection experiments that target a single trait [5] such as flowering time in our design.

The Late populations responded initially better to selection than the Early ones. It can be explained by directional epistasis, as expected for traits with a complex genetic architecture [26]. Note that the two initial inbreds *F*252 and *MBS* are early-flowering and adapted to temperate regions. As such, both have been submitted to intense selection for earliness and new mutations occuring in a background of coadapted early alleles are more likely to perturb existing combinations and to result in a late phenotype. In addition to the initial asymmetry between Late and Early populations, some Late populations displayed a plateau after generation G6 while Early populations continued to respond to selection over time. Under our Northern European climate, Late genotypes may be more prone to reach physiological limitations resulting in less efficient selection. This is precisely what we observed. After several generations of selection, the latest genotypes flowered so late that they were not able to produce mature seeds before the end of the season so that the contributors to the next generation were early plants within the Late populations. Therefore, the lack of response to selection observed within the Late populations in the second part of our experiment, from *G*7 to *G*16 reflects our lack of success to select for lateness. Such limits are common in DSEs and have been observed for example in mice body weight [33] and for maize protein and oil content [35].

Just as reported for DSEs conducted in other systems [30, 43, 44], hard sweeps [53] were observed between generations G0 and G6. Given our extremely small effective population size within families, this was expected. For example, the band for marker A1 was lost for all progenitors of the FL1 family as soon as generation G4 (Fig. 5). The same kind of pattern was observed for a couple of markers in strong LD with the *eIF*-4A gene. Interestingly, we previously proposed [51] that a cluster of tightly-linked genes including *eIF*-4A contribute to pleiotropic effects on plant height and leaf number in addition to flowering time. Such hypothesis is consistent with the pronounced pattern of linkage disequilibrium that we report here in this region (Fig. 4A). Note however that a strong LD pattern is also expected from selfing in small populations. Hence we did not merely observe causal relationships between marker polymorphims and flowering time, but rather the effect of selection at the genome-wide scale.

Estimates from the genome-wide genotyping of parent lines *F*252 and *MBS* with the 50K maize SNP microarray [54, 55] revealed a residual heterozygosity of about 1.9 % for *F*252, and 0.19 % for *MBS* over 29,000 SNPs mainly concentrated in genic regions. Our starting material was therefore characterized by a narrow genetic basis, even though our estimates are likely biased downards by undetected residual heterozygosity in gene-poor portions of the genome such as pericentromeric regions [56, 57]. With our molecular data we confirmed the greater variation of the *F*252 inbred, as compared with *MBS* (about one order of magnitude higher). Despite such a narrow genetic basis, standing variation for flowering time contributed to the response to selection. Consistently, we found a significant genetic variance component in F2 populations derived from crosses between one early- and one late-flowering plant from the initial *F*252 and *MBS* seed lots (data not shown). In *F*252, the measured genetic variance was similar to the one measured in an F2 population derived from a cross between an Early and a Late progenitor at generation G13, while in *MBS*, it was about half. Considering that close to a hundred flowering time QTLs have been reported in maize [45, 58], we would expect residual allelic variation for at most 2 of them in *F*252 and between zero and one in *MBS*. It is therefore remarkable that with so few initial genetic polymorphisms, phenotypic differences between Early and Late families reached around 150 degree days within 7 generations of selection, a range of variation comparable with the observed range of hybrids cultivated under Northern European conditions. Moreover, while *MBS* was initially an order of magnitude less polymorphic than *F*252, the measured phenotypic response to selection was similar in *F*252 and *MBS* populations (Fig. 2). Hence, residual heterozygosity of the initial inbreds can only partly explain the fast response to selection observed during the first generations in our DSEs.

While AFLPs markers are dominant and therefore not applicable to detect heterozygosity, our likelihood model relies on pedigree information to infer both homozygous and heterozygous genotypes. Using this framework, we observed a loss of initial polymorphism within a few generations at a rate in line with neutral predictions (given our effective population size). At G4, average heterozygote frequency has decreased from 0.5 to 0.04 in *MBS* and 0.07 in *F*252. At G7, all progenitors were homozygous for all markers. This finding is consistent with previous simulation results [46]. While we cannot exclude the role of initial polymorphisms in the response to selection (a gene space of 161Mb with a residual heterozygosity of 2 % corresponds to more than 3 million initial polymorphisms), our data indicate that initial polymorphisms are lost at the expected rate of one half per generation. Sources of polymorphism other than standing variation must therefore have contributed to sustain selection during 16 generations.

We did not identify new genetic or epigenetic variants in our setting while we expect roughly 800 of them per population within the gene space (considering 10 individuals per population, 16 generations, a gene space size of 161 Mb and a mutation rate of 3.10^−8^). We hence reached a paradox with on one hand, the maintenance of heritable phenotypic variation (Tables 1 and 2), and on the other hand, no evidence of new variation. In other words, we observed in our DSEs a dearth of polymorphisms associated with a sustained response to selection for flowering time. We propose two hypotheses to resolve this conflicting pattern: either we were not able to detect mutations that occured during the course of our experiment using our AFLP/M-SAP assays; or the the number of mutations was indeed low and maintenance of heritable variation through time is ensured by ‘conversion’ of epistatic variance (from either initial polymorphisms or new mutations) to additive variance [59-61].

Apparent lack of polymorphism could be an artefact of our technical choices and methods. First AFLPs and M-SAPs only reveal a sample of existing polymorphisms (the ones associated with restriction sites) and are non randomly distributed. They tend to cluster in non recombining regions [62]. Whether these regions are less likely to reveal de novo mutations is however questionable. Alternatively, the observed phenotypic variation may merely be sustained by unstable changes in DNA methylation status that we were unable to detect. Indeed, our method to reconstruct AFLP/M-SAP genotypes from the band patterns (phenotypes) and the pedigrees was fairly stringent. Polymorphisms that appeared in one progenitor without being mendelian transmitted to its offspring were typically counfounded with experimental errors and discarded. Note that even if those polymorphisms had been considered, it would have been impossible to test their association with flowering time variation because of lack of power. However when referring to the litterature, we overall expect a limited contribution of unstable methylation changes to phenotypic changes. Indeed, studies in maize have revealed that the vast majority of differentially methylated regions including pure epigenetic variants are mostly mendelian inherited [63]. Finally, we must keep in mind that M-SAP methodology only reveals DNA methylation polymorphisms and not histone methylation polymorphisms. The former have been associated with quantitative variation of traits in *Arabidopsis thaliana* including flowering [64], and results suggest that the latter may be an important determinant of natural variation [65]. Along the same line, our methodology did not allow detection of structural variation caused by the insertion-deletion of transposable elements. Such elements are known to be important contributors of phenotypic variation in crops [66, 67].

Alternatively, conversion of epistatic to additive variance could explain the significance of the response to selection through time even with a limited number of mutations. This "make a lot with few" hypothesis relies on the process of fixation of initial polymorphisms and the fact that subtle within-family genetic background changes may reveal cryptic genetic variation [68]. There are two possible mechanisms underlying this phenomenon. First, a loss-of-function mutation in a canalization gene can reveal hidden genetic or phenotypic variation, like mutations in the chaperone protein Hsp90 [69, 70]. Second, a mutation introduced in different genetic backgrounds can reveal epistatic effects of the background that range from complete suppression to enhancement of the mutant phenotype, as evidenced in *Drosophila* for several mutations in homeotic genes (reviewed in [10]) but also in maize for the *tb1* mutation that control branching during early development [71, 72]. Interestingly, just like some examples above concern developmental genes, we found that flowering time variation in our DSEs was intimately linked with changes in the timing of floral transition [51], a shift determined during early maize development. Strong epistatic variations hidden at a population level but revealed by a combination of strong drift and selection in our experimental setting may explain elevated mutational variance [46] as compared with more traditional settings [10]. Our AFLP/M-SAP assays clearly present limitations in the detection of new mutations. Only genome-wide exploration of mutations and epimutations using high throughput technologies will offer enough power to distinguish between those two explanations for the lack of polymorphism found in our DSEs.

## Conclusions

We describe here two experiments that consisted in 17 generations of divergent selection for flowering time in maize, starting from a single inbred line seed lot. We observed a sustained response to selection in both directions. In the Late populations a physiological limit was reached within a few generations, but we showed evidence for the maintenance of heritable genetic variation. Residual heterozygosity in the initial inbreds can partly explain the observed responses as evidenced by M-SAP and AFLP markers. Their allelic segregation throughout the DSEs’ pedigrees indeed revealed a strong association with flowering time variation. Their fast fixation resulted in the emergence of strong genetic differentiation between the Early and the Late populations. During the first 7 generations, we found no evidence of new polymorphisms arisen by mutation. Whether unexplored sources of polymorphism or strong epistatic interactions account for the observed dearth of polymorphism remains an unresolved issue that will deserve further investigations. Altogether we produced a material of choice to study the effects of those mutations. After 17 generations of selection, we produced sets of phenotypically differentiated genotypes that share a common genetic background inherited from the inbred parent. The challenge now will be to decipher differences in the developmental sequence that were targeted by selection to modify flowering time.

## Methods

### Plant material and experimental design

Starting from the commercial inbred lines *F252* and *MBS847* (*MBS*), we have conducted two Divergent Selection Experiments (DSEs) for flowering time. From initial seed lots of the two inbred lines, we derived four populations: an Early *F252* population, a Late *F252* population, an Early *MBS* population and a Late *MBS* population by successively selecting and selfing the earliest and the latest plants at each generation. The propagation method is equivalent to single seed descent except that some plants can have more than one descent, and some can have less due to selection. A progenitor designates a plant belonging to the DSEs and its corresponding seed lot obtained by selfing. The selection procedure for the seven first generations as well as the resulting pedigrees are described in details in [46]. Briefly, selected progenitors at generation *g* were selfed to produce 100 seeds which were evaluated into 4 rows of 25 seeds in a randomized block design. Selection of progenitors was performed first by choosing the 3 earliest plants within each row of Early progenitors (total of 12 plants per progenitor within Early populations) and the 3 latest plants within each row of Late progenitors (total of 12 plants per progenitor within Late populations). Second, only the 10 most extreme progenitors (Early and Late) with the highest seed weights were retained to constitute the generation *g*+1. The resulting pedigrees at Generation G6 contained 62 progenitors except the Late *MBS* which contained 63 progenitors (Fig. 5). We carried out the selection experiment from generation *G*7 to *G*16 following the same procedure. The resulting pedigrees from *G*0 to *G*16 are presented in Fig. 1.

### Evaluation of all progenitors from *G*0 to *G*6 in 2004 and 2005

Of the 249 selected progenitors (from generation *G*0 to *G*6), S2 seeds (produced by two generations of selfing) from 229 progenitors were evaluated in a 2 years field trial (in 2004 and 2005) at Gif-sur-Yvette (France) following the procedure described in [46]. Controls were S2 seeds produced from the *F*252 and *MBS* initial seed lots. Briefly, progenitors were evaluated in randomized block design and female flowering time was recorded as the date (in days after july 1st) at which 50 % of plants within a plot were silking. For the present paper, we converted flowering dates in thermal time following [73] (with parameter values Tb 6 and To 30). We estimated genotypic data after correcting for fixed year, and block within year effects [46]. Analyzes were carried out using the R software [74].

### Response to selection from G0 to G16

As described above, we recorded at each generation the flowering dates of 12 plants per progenitor within Early and within Late poualtions. After transformation into thermal time and correction for the block effects, data collected from generations *G*0 to *G*16 were analyzed separately for each DSE. Within each data set, we supposed that variations for flowering date *Z* could be decomposed into a fixed year effect for all progenitors, a linear response component that depended on the population (Late, Early, Control), and a residual.

(1) $$ Z_{ijklm} = Year_{i} + b_{j} \cdot gener_{i} + \epsilon_{ijklm}  $$

where *i* stands for the year and corresponding generation of selection, *j* for the population, *k* for the family within population, *l* for progenitor within family, and *m* for the plant measurements within progenitor. We used equation (1) to correct the data for the fixed year effect. In order to verify the validity of this approach we compared the resulting corrected flowering time estimated from *G*0 to *G*6 with the ones obtained from the *G*0−*G*6 complete evaluation trials (in 2004 and 2005). As shown in Additional file 1: Figures S1 and S2, there was an extremely high correlation between the two measures (*r*=0.83 for *F*252 and *r*=0.88 for *MBS*, p-values lower than 2.2*e*−16 in both cases). We therefore used yearly measurements of the most extreme plants to analyze the response to selection over 16 generations. We corrected values $Y_{\textit {ijklm}}=Z_{\textit {ijklm}} - \hat {Year}_{i}\phantom {\dot {i}\!}$ to analyze separately the response to selection for each independent family. Note that we did not exactly measure the same trait as the one measured in the evaluation trial [46], i.e. extreme values *versus* average, which explains why the previously nearly significant reponse to selection in Early *MBS* [46] became significant here from generation *G*0 to *G*6.

The linear component *b*_*jk*_ of the within-family response to selection was estimated using the following linear model:

$$Y_{ijklm} = \mu_{0} + b_{jk} \cdot gener_{i} + \epsilon_{ijklm} $$

where *μ*_0_ is the intercept corresponding to the average flowering time at generation *G*0. In order to compare the two DSEs, data used to plot Fig. 2 were standardized by the residual deviation of the control line.

At each generation, the genetic variance between progenitors of the same family was measured using the following mixed linear model:

$$Y^{(ijk)}_{lm} = \mu^{(ijk)} + G^{(ijk)}_{l} + \epsilon^{(ijk)}_{lm} $$

where *Y* is the corrected flowering time of plant *m* from progenitor *l*. Broad sense heritabilities $H^{2}_{\textit {ijk}} = \frac {var(G)_{\textit {ijk}}}{var(G)_{\textit {ijk}} + var(\epsilon)_{\textit {ijk}}}\phantom {\dot {i}\!}$ measured the part of the phenotypic variance that can be attributed to differences among progenitors within family. Note that the residual variance *var*(*ε*) also encompassed a genetic component corresponding to the genetic variance between offspring from the same progenitor. Because markers were genotyped only progenitors from generation *G*0 to *G*6, separate analyses for the response to selection were conducted between *G*0 and *G*6, and between *G*7 and *G*16. Within-familiy heritabilities were estimated, median and maximum values are reported in Table 1 and Table 2. Full distributions are given in Additional file 1: Figures S3 and S4.

### AFLP and M-SAP phenotyping

In 2004, we extracted genomic DNA from leaf material of 3 S2 plants per selected progenitor (from generation *G*1 to generation *G*6). To investigate both mutations and epimutations, we performed Methyl-Sensitive Amplification Polymorphism (M-SAP) technique [75], a modified version of the Amplified Fragment Length Polymorphism (AFLP) [76]). We used EcoRI as rare cutter enzyme and, alternatively one of the two isoschizomers HpaII and MspI. Both HpaII and MspI recognize the 5‘-CCGG-3’ restriction site but differ in their sensitivity to methylation. Hence different methylation state of the internal cytosine at the 5‘-CCGG-3’ restriction sites results in different cleavage by the isoschizomers, generating variable PCR band profiles between the two digests. We also used a more classical AFLP procedure using the EcoRI/MseI digests. The AFLP and the M-SAP procedures were performed according to [76] with minor modifications. Briefly, 250 ng of genomic DNA was digested with three digestion systems: EcoRI and MseI, EcoRI and MspI, EcoRI and HpaII in a final volume of 25 *μ*L following the provider directions (BioLabs). After inactivation (15 min at 65 °C), preparation and ligation of the adaptors were performed following [77]: EcoRI adaptors (5‘CTCGTAGACTG CGTACC3’ and 5‘AATTGGTACGCAGTCTAC3’), MseI adaptors (5‘GACGACGAGTCCTGAG3’ and 5‘TACTCAGGACTCAT3’), MspI/HpaII adaptors (5‘GACGATGAGTCTAGAA3’ and 5‘CGTTCTAGACTCATC3’). A ligation mixture of 15 *μ*L was added to the digested DNA. The resulting reaction mix was incubated for 16 hours at room temperature. This was followed by a pre-amplification step using primers complementary to the adaptors with an additional selective base (EcoRI+A, MseI+C, MspI/HpaII+C) and performed in a total volume of 50 *μ*L containing 1x PCR buffer, 5 *μ*L of the diluted digested-ligated DNA (1:10 in water), 0.4 M of each primer, 0.2 mM dNTPs, 2 mM MgCl2, and 2 units of Taq DNA polymerase. Cycling parameters were: 94 °C/2 min followed by 20 cycles of 94 °C/30 s, 56 °C/1 min, 72 °C/1 min and a final cycle of 72 °C/5 min. Selective amplifications were performed using 3 additional selective bases for a total of 8 selective primers per enzyme and 64 (8x8) combinations of primers per enzymes pairs: EcoRI (E+AAC; E+AAG;E+ACA; E+ACC; E+ACG; E+ACT; E+AGC; E+AGG), MseI (M+CAA; M+CAC; M+CAG; M+CAT; M+CTA; M+CTC;M+CTG: M+CTT), MspI/HpaII (MH+CGG; MH+CTA; MH+CGC; MH+CGT; MH+CAC; MH+CTG; MH+CTC; MH+CTT). Amplification mixture was performed as described above from 5 *μ*L of the diluted preamplification products (1:10 in water). The cycle conditions were 94 °C/2 min, 12 touchdown cycles of 94 °C/30 s, 65 °C/1 min (-0.7 °C each cycle), 72 °C/1 min, followed by 23 cycles of 94 °C/30 s, 56 °C/30 s, and 72 °C/1 min, and final extension at 72 °C/5 min. Amplification products were diluted (1:20) in loading buffer (94 % formamide, 0.5 mg/ml bromophenol blue), and migrated, after denaturation (5 min at 95 °C) in a 40 cm 5.5 % denaturing (6 M urea) long range acrylamide gel (BMA, Rockland, ME, USA) in 1x TBE. The electrophoresis was performed in a LI-COR DNA analyzer (LI-COR, Lincoln, NE, USA) at 2,000 V for 6 h at 50 °C, using the LI-COR 50–700 bp size standard as internal ladder. The AFLP and M-SAP techniques were first applied to 4 progenitors, an Early and a Late from the F252 and MBS pedigrees. The primer combinations (3 selective bases) that exhibited the highest level of polymorphism between Late and Early progenitors within F252 and MBS were retained for further analyses. In total, we selected 6 primer combinations for the EcoRI and MseI digests: 4 to be applied to the progenitors derived from F252 (E+ACC/M+CAC; E+AGG/M+CAG; E+ACT/M+CTA; E+AGG/M+CTT) and 2 to be applied to the progenitors derived from MBS (E+ACC/M+CAG; E+ACA/M+CTG). We selected 7 common primer combinations for the EcoRI and MspI/HpaII to be applied to progenitors derived from both F252 and MBS (E+AAC/MH+CTA; E+ACG/MH+CGC; A+ACT/MH+CGT; E+ACT/MH+ CAC; E+AAG/MH+CAC; E+ACA/MH+CAC; E+ACT/MH+CTC). In order to validate our AFLP/M-SAP procedures on DNA bulks, we performed 3 DNA extractions from 3 S2 plants to represent a single mother progenitor. We assessed the reliability of employing bulks by comparing profiles obtained with 3 independent S2 plants loaded separately on a gel, with profiles obtained from bulks of S2 plants. Bulks were either performed by mixing the 3 DNAs before performing the AFLP/M-SAP assays or by combining in equal quantity 3 amplification products on a single lane of the acrylamide gel. We repeated this test by bulking artificially all possible genotype combinations (0-0-0), (1-0-0), (1-1-0), (1-1-1), with 0 and 1 corresponding respectively to the absence and presence of a band. We obtained reliable results with the bulking of PCR products, i.e. the presence of a band in one reaction/two or three reactions was reliably detected in the bulk. We therefore employed this procedure for the subsequent genotyping, reducing by 3 the number of gels generated. In total, we generated 249 progenitors × 10 primer combinations (11 and 9 respectively for F252 and MBS) for a total of 2490 phenotyping. Two persons visually scored bands and only concurring AFLP/M-SAP phenotypes were retained for further analyses. We categorized the M-SAP markers as follows: markers exhibiting the same polymorphism patterns with the two digestion systems EcoRI/MspI and EcoRI/HpaII were considered sequence-based polymorphisms; markers polymorphic only with EcoRI/MspI but not with EcoRI/HpaII were considered as located in a methylation region; markers polymorphic only with EcoRI/HpaII but not with EcoRI/MspI were considered as methylation-based polymorphisms.

### Genotype inferences and statistical analyses

We produced a matrix of AFLP and M-SAP phenotypes in the form of presence-absence of a band at each marker for each progenitor of the pedigrees. While these markers possess several advantages in terms of cost and time, they also present some limitations in part due to our specific experimental design. We identified three of them: first, both AFLPs and M-SAPs are dominant markers and provide no information on the level of heterozygosity, but information about the pedigrees may be used to infer heterozygotes; second, we used as a source of DNA a bulk of three S2 plants from each progenitor of the pedigrees, thereby generating indirect access to the original genotypes; third, both types of markers are subject to various sources of experimental biases due, for instance, to allele competition during PCR or homoplasy that need to be considered. To account for all three sources of genotype miscalling, we developed a likelihood model and utilized a parcimony algorithm to search for the matrix of genotypes with the highest likelihood at every given AFLP locus. In Additional file 2, we describe the corresponding procedure as well as the likelihood function that calculates, for a given locus, the probability of each genotype given the marker phenotype for all progenitors of the pedigrees at all generations. We combined inferred genotypes across all loci to perform a Linear Discriminant Analysis (LDA). In LDA, axes are defined by a linear combination of markers that best discriminate the progenitors. Outputs provide the percentage of variation explained by the axes. We also perfomed analyses of molecular variance (AMOVA, [52]) from allele frequencies at each generation, within population (Late, Early) and family (≈ 2 families per population) as grouping factor. Significance was assessed by permutation tests using the Vegan R package [78].

### Association mapping

Within each DSE, the values of the additive (*a*) and dominance (*d*) effects associated with each candidate locus were estimated by linear regression:

$$G_{il} = \mu_{i} + a \cdot x_{il} + d \cdot y_{il} + \epsilon_{il} $$

where *G*_*il*_ is the breeding value of progenitor *l* at generation *i*, *μ*_*i*_ is the average flowering time calculated over all progenitors at generation *i*, and *x*_*il*_ and *y*_*il*_ are indicator variables of the allelic status of progenitor *l* at the candidate locus. The pair (*x*_*il*_,*y*_*il*_) was (−1,0) when the band was absent, (0,1) for heterozygotes at the marker locus, and (1,0) for homozygotes with two copies of the band. As described in [46], we tested the significance of the association between flowering time variation and the segregation of alleles at the AFLP markers by simulating a null distribution for the additive (*a*) and dominance (*d*) parameters considering simulated genotypes and observed phenotypic values. Genotypes were simulated by dropping two alleles (presence or absence) throughout the pedigrees considering heterozygous ancestors at generation *G*0. At each generation, the genotype of each progenitor was drawn at random knowing the genotype of its parent and assuming Mendelian inheritance and no reversion. Probabilities of observed *a* and *d* among 10,000 simulations were determined (P-values). For each DSE, we performed a global analysis and two separate ones, within Early and within Late populations.

### Linkage disequilibrium

At each marker, within genotype band frequency (0, 0.5 or 1) was deduced from the AFLP genotypes. Linkage disequilibrium (LD) between markers was computed as the square of the correlation coefficient between genotype band frequencies, *r*^2^. Notice that *r*^2^ measures the association between alleles at two different loci that can be due either to gametic LD or to departure from random mating [79]. Because all progenitors were obtained by selfing, we assumed that the LD component due to departure from random mating was homogeneous throughout the genome. Hence, *r*^2^ only depended upon allele frequencies and genetic distance between loci. Significance was assessed by random permutations of the genotypes between progenitors. We assumed that LD should decrease linearly with genetic distance. The LD matrix was used to order the markers following the same rationale as for building-up genetic maps. In other words, first LD linkage groups were formed using pairwise LD p-values. Second, within each linkage group, markers were automatically ordered using functions similar to the MapMaker’s *compare* and *riffle* functions [80], and an iterative procedure as described in [54]. The statistics used to compare possible orders was based on transformation of the LD matrix into ranked value. Under the best order, off-diagonal elements sharing the same number of intervals should have similar LD ranks. Once ordered, LD map distance between adjacent markers was computed as $d_{\textit {ij}} = - log(r^{2}_{\textit {ij}})\phantom {\dot {i}\!}$ and was considered additive.

## Availability of supporting data

The data set(s) supporting the results of this article is(are) available in the DRYAD repository [81], http://dx.doi.org/10.5061/dryad.7bj60.

## Additional files

Additional file 1
**Additional Figures.** Distribution of within families heritabilities in each population of the DSE, and correlations between flowering time measured in the evaluation trials and in the DSEs.

Additional file 2
**Additional Methods.** Description of the likelihood method used to infer the AFLP genotypes from band scorings.
