# Supplementary material for: Dearth of polymorphism associated with a sustained response to selection for flowering time in maize
Source: BMC Evol Biol. 2015 Jun 7;15:103. doi: 10.1186/s12862-015-0382-5 (PMC4458035; doi:10.1186/s12862-015-0382-5)
Supplement: Additional file 1 — Additional Figures. Distribution of within families heritabilities in each population of the DSE, and correlations between flowering time measured in the evaluation trials and in the DSEs. [file 12862_2015_382_MOESM1_ESM.pdf]

Dearth of polymorphims associated with a sustainable response to selection for flowering time in maize

Additional Figures

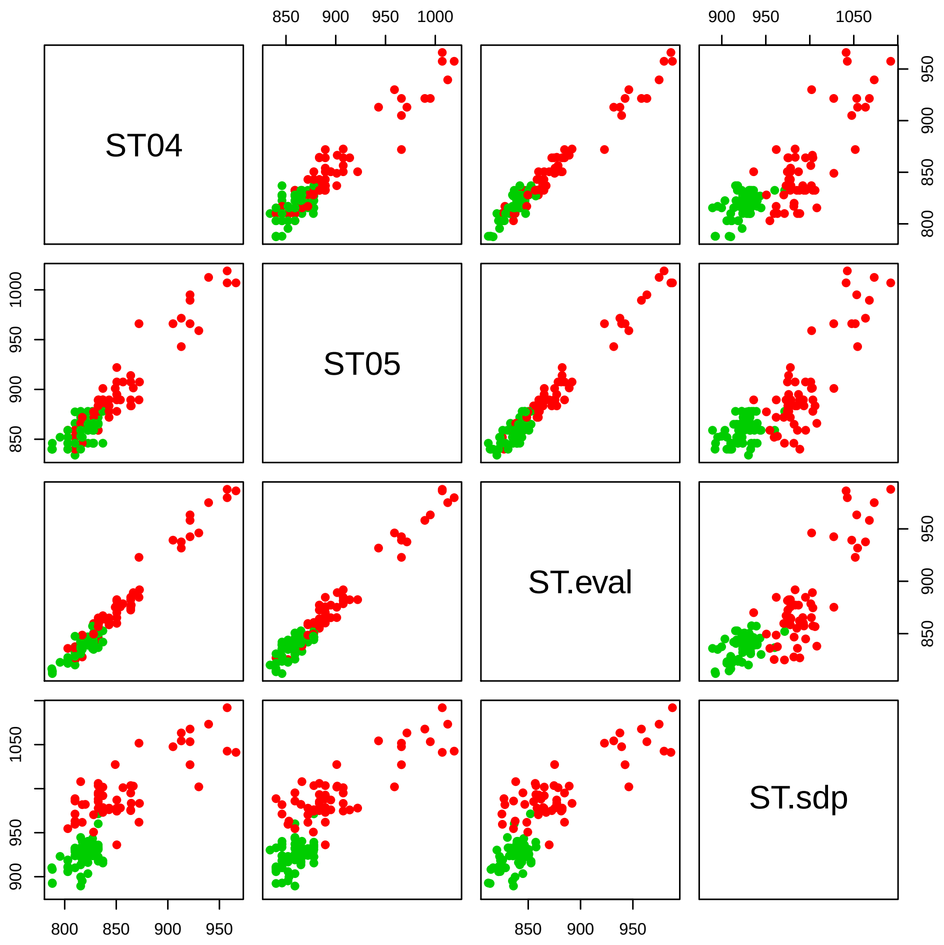

Figure S1 – **Correlation between evaluation trial and yearly measurements in the $F252$ DSE.** Each point correspond to one DSE progenitor between generations $G1$ and $G6$. S2 plants were evaluated in a field trial in 2004 (ST04) and 2005 (ST05) with two plots per progenitor in a random block design. The average flowering time of each plot was recorded and corrected for block effects. After correction for the year effect, we obtained predictions for the average flowering time (ST.eval). Data recorded yearly in the DSE between 1997 (G1) and 2002 (G6) are extreme values for flowering time recorded on 12 plants per progenitor (12 latests in te Late populations and 12 earliest in the Early populations). After correction for block within year and year effects, we obtained the DSE predictions for flowering time (ST.sdp). Flowering time is measured in thermal time. Early progenitors are in green. Late progenitors are in red.

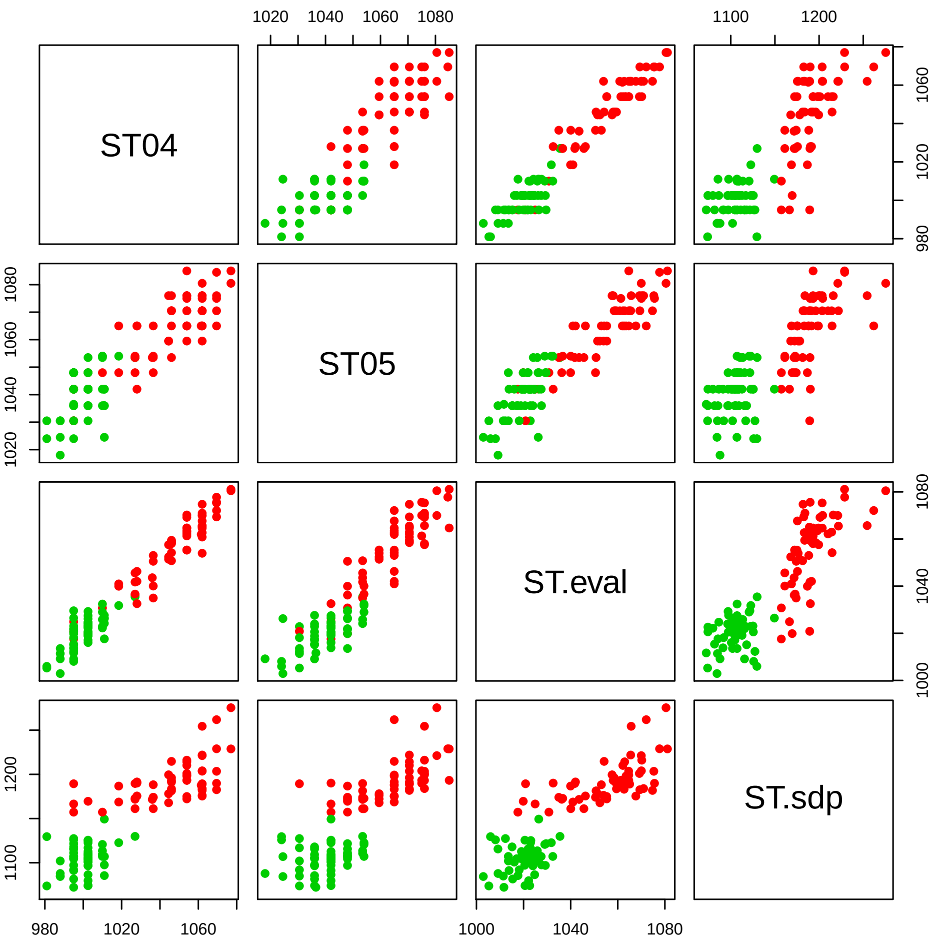

Figure S2 – **Correlation between evaluation trial and yearly measurements in the** MBS **DSE.** Each point correspond to one DSE progenitor between generations $G1$ and $G6$. S2 plants were evaluated in a field trial in 2004 (ST04) and 2005 (ST05) with two plots per progenitor in a random block design. The average flowering time of each plot was recorded and corrected for block effects. After correction for the year effect, we obtained predictions for the average flowering time (ST.eval). Data recorded yearly in the DSE between 1997 (G1) and 2002 (G6) are extreme values for flowering time recorded on 12 plants per progenitor (12 latests in te Late populations and 12 earliest in the Early populations). After correction for block within year and year effects, we obtained the DSE predictions for flowering time (ST.sdp). Flowering time is measured in thermal time. Early progenitors are in green. Late progenitors are in red.

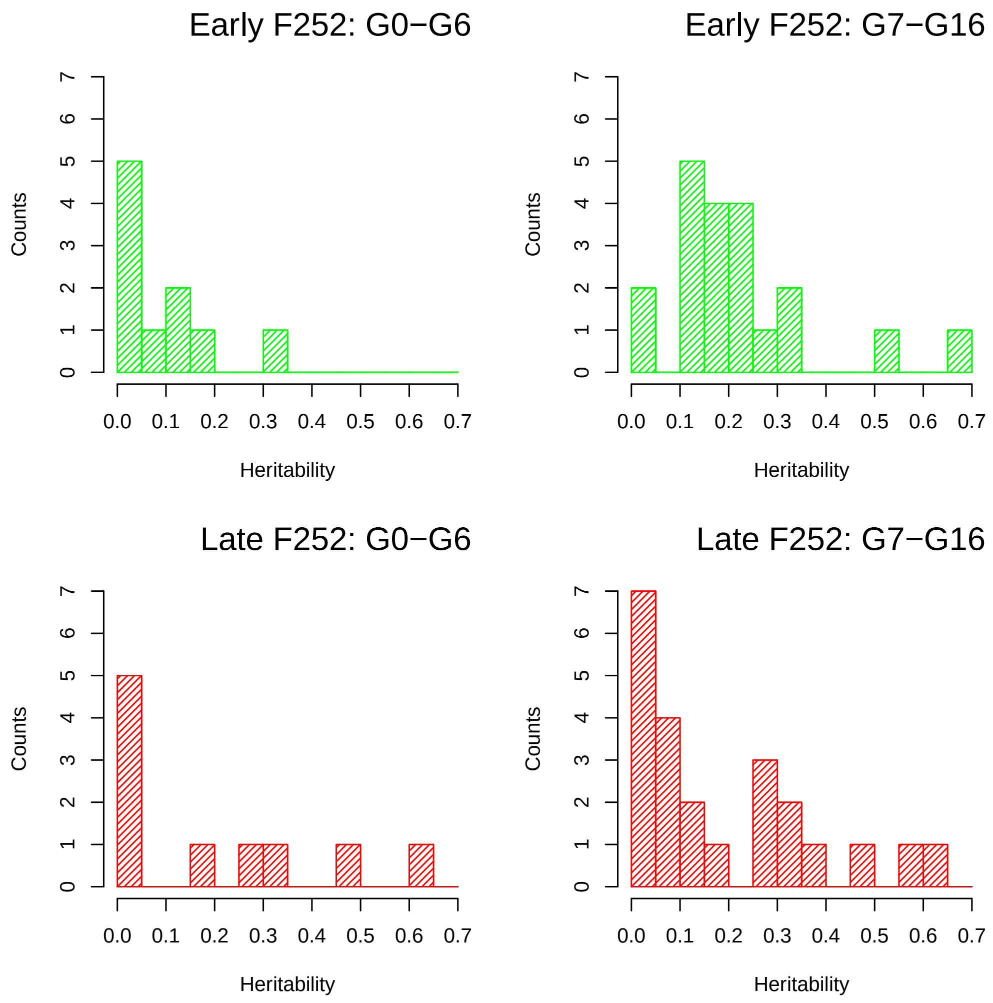

FIGURE S3 – **Distribution of heritabilities for flowering time in the $F252$ DSE.** Heritabilities were measured within each family at each generation as the ratio of the genetic variance between progenitors over the total phenotypic variance. Data from generations $G0 - G6$ and generations $G7 - G16$ are represented separately in each population.

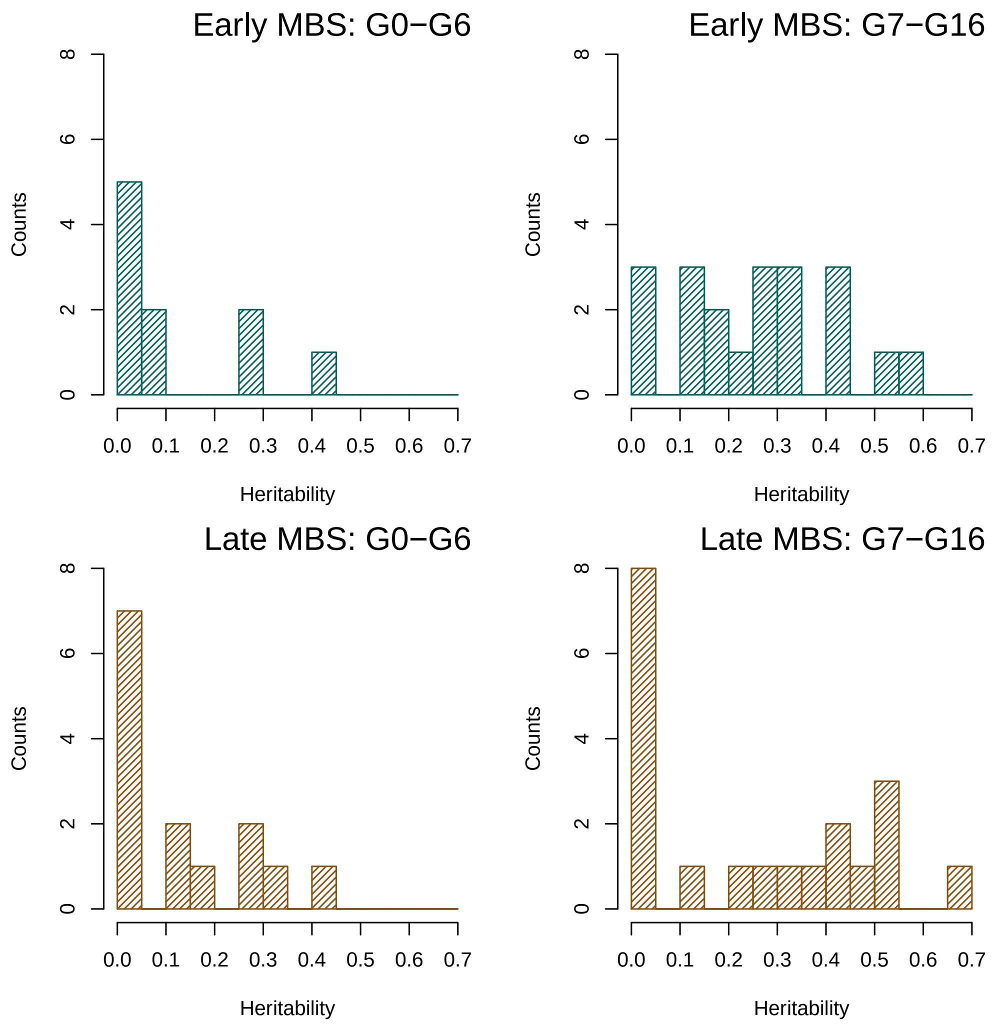

Figure S4 – **Distribution of heritabilities for flowering time in the MBS DSE.** Heritabilities were measured within each family at each generation as the ratio of the genetic variance between progenitors over the total phenotypic variance. Data from generations $G0 - G6$ and generations $G7 - G16$ are represented separately in each population.
