# Supplementary material for: Dearth of polymorphism associated with a sustained response to selection for flowering time in maize
Source: BMC Evol Biol. 2015 Jun 7;15:103. doi: 10.1186/s12862-015-0382-5 (PMC4458035; doi:10.1186/s12862-015-0382-5)
Supplement: Additional file 2 — Additional Methods. Description of the likelihood method used to infer the AFLP genotypes from band scorings. [file 12862_2015_382_MOESM2_ESM.pdf]

Dearth of polymorphims associated with a sustainable response to selection for flowering time in maize

Inferring AFLP genotypes from phenotypes and relatedness relationships

We have produced a matrix of AFLP phenotypes in the form of presence-absence of a band at each marker for each individual of the genealogy. While these markers possess several advantages in terms of cost and time, they also present few limitations linked or not to our specific experimental design. We identified three of them : first, AFLPs are dominant markers and provide no information on the level of heterozygosity, but information about the genealogy may be used to infer heterozygotes ; second, we used as a source of DNA a bulk of three S2 (issued from two generations of selfing) individuals issued from each individual of the genealogy, thereby generating indirect access to the original genotypes ; third, AFLPs are subject to various sources of experimental biases due, for instance, to allele competition during PCR or homoplasy that need to be considered. To account for all three sources of genotype miscalling, we developed a likelihood model and utilized a parcimony algorithm to search for the matrix of genotypes with the highest likelihood at every given AFLP locus. Below we describe the likelihood function that calculates, for a given locus, the probability of each genotype given the AFLP phenotype for each individual of the genealogy at each generation. This function considers (i) the genealogical information, (ii) the generations of selfing, (iii) the experimental errors associated with AFLPs.

1 Heredity

Let $X_{g,i} \in \{AA, Aa, aa\}$ be the random variable associated to the genotype of individual i at generation g at the AFLP biallelic locus. Allele A (presence of a band) is dominant over allele a (absence of a band). $X_{g,i}$ can be associated to a probability law :

$$\vec{\pi}(X_{g,i}) = \{P(X_{g,i} = AA), P(X_{g,i} = Aa), P(X_{g,i} = aa)\}'$$

Conditionally to the parent $\mathcal{A}_{g,i}$, we have, for any realization $x_o \in \{AA, Aa, aa\}$:

$$P(X_{g,i} = x_o) = \sum_{x_a \in \{AA,Aa,aa\}} P\left(X_{g,i} = x_o / X_{g-1,\mathcal{A}_{g,i}} = x_a\right) \cdot P\left(X_{g-1,\mathcal{A}_{g,i}} = x_a\right) \qquad (1)$$

Considering that the individuals are reproducing by selfing at each generation, that mutations occur during meiosis at a rate μ per individual per generation and can lead with equal probability to the loss ($A \to a$) or the gain ($a \to A$) of a band, and that the strength of selection introduces an advantage s to the heterozygotes, the frequency of selected genotypes x_o among the progenies of parent x_a is

$$p_{x_o/x_a} = \frac{f(x_o/x_a)w_{x_o}}{\sum_{x_k \in \{AA,Aa,aa\}} f(x_k/x_a)w_{x_k}}$$

where $f(x_k/x_a)$ is given by mendelian inheritance taking into account selfing and mutations. Selection occurred among the adult progenies, with relative selection coefficients $w_{AA} = w_{aa} = 1$ and $w_{Aa} = 1 + s$. We established the (3×3) matrix T of probabilities of $X_{g,i}$ given $X_{g-1,\mathcal{A}_{g,i}}$ as indicated in the table below :

	parent		
offspring	AA	Aa	aa
AA	$\frac{(1-\mu)}{1+\mu s}$	$\frac{1}{4+2s}$	0
Aa	$\frac{\mu(1+s)}{1+\mu s}$	$\frac{1+s}{2+s}$	$\frac{\mu(1+s)}{1+\mu s}$
aa	0	$\frac{1}{4+2s}$	$\frac{1-\mu}{1+\mu s}$

so that,

$$P\left(X_{g,i} = x_o / X_{g-1,\mathcal{A}_{g,i}} = x_a\right) = T[x_o, x_a] \tag{2}$$

Using (1) and the T matrix,we have :

$$\vec{\pi}(X_{g,i}) = T \cdot \vec{\pi}(X_{g-1,\mathcal{A}_{g,i}}) \tag{3}$$

2 Modeling AFLP phenotypes

Let $Y_{g,i} \in \{0,1\}$ be the random variable associated to the AFLP phenotype of individual i at generation g, and $y_{g,i}$ the observation. Because of both bulk genotyping and experimental errors, the AFLP phenotype does not translate directly into the genotype.

2.1 Bulk genotyping

For genotyping, each individual S_0 of the genealogy has been selfed once to produce 25 S_1 individuals, also selfed with seeds (S_2) collected in bulk. We used DNAs from three S_2 plants to infer the genotype of the original individual. This procedure may lead to ascertainment bias. For instance, three S_2 plants generated from a S_0 heterozygote may by chance be homozygotes for the a allele leading to the absence of the band. This may occur with a probability 0.5 at each generation of selfing (0.25 in S_2). Let

$$p_b = P\left(Y_{g,i} = 0 / X_{g,i} = Aa\right) \tag{4}$$

In the bulk of 3 S_2 plants, we have $p_b < 0.05$. Because we performed many independent bulks, we nevertheless consider this eventuality by modeling p_b as a random variable following an exponential distribution of parameter $\lambda_b = 1/0.05 = 20$.

2.2 Experimental bias

Experimental biases may occur if primers attach preferentially to one of the allele causing differences among genotypes and/or alleles within a genotype in heterozygotes, or when there is homoplasy, i.e. presence of non-allelic bands of the same size. We considered those as random events associated the probability p_a and p_h, which designate the probability of not observing a band (0) in a genotype possessing at least one allele A (p_a) and the probability of observing a band (1) in an homozygote aa genotype (p_h), respectively.

2.3 AFLP phenotypes

Considering all possible sources of error (bulk genotyping and experimental biases), the table below gives the associated probabilities of the AFLP phenotype knowing its genotype $P\left(Y_{g,i}/X_{g,i}\right)$

	X		
Y	AA	Aa	aa
0	p_a	$(1-p_b)\cdot p_a + p_b\cdot(1-p_h)$	$1-p_h$
1	$1-p_a$	$(1-p_b)\cdot(1-p_a) + p_b\cdot p_h$	p_h

3 Likelihood function

We have described above how to estimate the probability of a phenotype knowing the genotype of the individual and also how to calculate a vector of probability of the 3 genotypes knowing the genotype of its parent. From there we were able to estimate the likelihood of a genotype of the genealogy knowing its AFLP phenotype and its parent.
The parameters of model are :
– $\theta_{hered} = (\mu, s)$ that relate to heredity.
– $\theta_{exp} = (p_b, p_a, p_h)$ that relate to the experiment.
The known information is $\mathcal{A}_{g,i}$ the parent of each individual of the genealogy. The random variables are $Y_{g,i}$ and $X_{g,i}$. Using conditional probabilities, we have

$$P\left(X_{g,i}/Y_{g,i}, \theta_{exp}, \theta_{hered}, X_{g-1,\mathcal{A}_{g,i}}\right) = \frac{P\left(X_{g,i}, Y_{g,i}, \theta_{exp}, \theta_{hered}, X_{g-1,\mathcal{A}_{g,i}}\right)}{P\left(Y_{g,i}, \theta_{exp}, \theta_{hered}, X_{g-1,\mathcal{A}_{g,i}}\right)} \tag{5}$$

Because the AFLP phenotype knowing the genotype does not depend on the parental genotype, and because θ_{hered} and $X_{g-1,\mathcal{A}_{g,i}}$ are independent, we have

$$\begin{aligned}
&P\left(X_{g,i}, Y_{g,i}, \theta_{exp}, \theta_{hered}, X_{g-1,\mathcal{A}_{g,i}}\right)\\
&= P\left(Y_{g,i}, \theta_{exp}/X_{g,i}\right) \cdot P\left(X_{g,i}/\theta_{hered}, X_{g-1,\mathcal{A}_{g,i}}\right) \cdot P\left(\theta_{hered}\right) P\left(X_{g-1,\mathcal{A}_{g,i}}\right)\\
&= P\left(Y_{g,i}/\theta_{exp}, X_{g,i}\right) \cdot P\left(X_{g,i}/\theta_{hered}, X_{g-1,\mathcal{A}_{g,i}}\right) \cdot P\left(\theta_{exp}\right) \cdot P\left(\theta_{hered}\right) \cdot P\left(X_{g-1,\mathcal{A}_{g,i}}\right)
\end{aligned}$$

Similarly, the denominator of (5) writes :

$$\begin{aligned}
&P\left(Y_{g,i}, \theta_{exp}, \theta_{hered}, X_{g-1,\mathcal{A}_{g,i}}\right)\\
&= P\left(Y_{g,i}/\theta_{exp}, \theta_{hered}, X_{g-1,\mathcal{A}_{g,i}}\right) \cdot P\left(\theta_{exp}\right) \cdot P\left(\theta_{hered}\right) \cdot P\left(X_{g-1,\mathcal{A}_{g,i}}\right)\\
&= P\left(\theta_{exp}\right) \cdot P\left(\theta_{hered}\right) \cdot P\left(X_{g-1,\mathcal{A}_{g,i}}\right) \cdot\\
&\quad \textstyle\sum_{x_o \in \{AA, Aa, aa\}} P\left(Y_{g,i}/X_{g,i} = x_o, \theta_{exp}\right) \cdot P\left(X_{g,i} = x_o/\theta_{hered}, X_{g-1,\mathcal{A}_{g,i}}\right)
\end{aligned}$$

Therefore, we have,

$$P\left(X_{g,i}/Y_{g,i}, \theta_{exp}, \theta_{hered}, \mathcal{A}_{g,i}\right) = \frac{P\left(Y_{g,i}/X_{g,i}, \theta_{exp}\right) \cdot P\left(X_{g,i}/\theta_{hered}, X_{g-1,\mathcal{A}_{g,i}}\right)}{\sum_{x_o} P\left(Y_{g,i}/X_{g,i} = x_o, \theta_{exp}\right) \cdot P\left(X_{g,i} = x_o/\theta_{hered}, X_{g-1,\mathcal{A}_{g,i}}\right)} \tag{6}$$

Equation (6) can be computed using the models described above. Furthermore, knowing the parent of each individual of the genealogy, conditional probabilities in (6) do not use any extra information and they are independent. Therefore, we extended this equation to all individuals of the population in order to capture the genealogical information contained in related individuals. The population is characterized by a combination of genotypes whose likelihood is the product of the likelihood of each individual genotype

$$\mathcal{L} = \prod_{g,i} P\left(X_{g,i}/Y_{g,i}, \theta, \mathcal{A}_{g,i}\right) \tag{7}$$

3.1 Parcimony algorithm

A population (genealogy) contains on average about 60 individuals with 3 possible genotypes AA, Aa, aa and therefore 3^{60} ($\approx 4 \times 10^{28}$) genotype combinations to explore. We developed a parsimony algorithm in order to obtain a limited set of matrix of genotypes and determine the

one with the highest likelihood from (6). In order to do so, we set up a backward-algorithm from generation 6 to generation 0 (most recent common ancestor of the whole population) that incorporates combinations of genotypes having the highest likelihood knowing the parameters θ.
Going backward in time, we considered each node of the genealogy as a $(k+1)$-uplet encompassing a parent p_i and its k offsprings o, $(p_i, o_{i1}, ..., o_{ik})$. Without prior information, the corresponding number of combinations at each node is $3^{(k+1)}$. At each generation and node, our algorithm consists in (1) calculating the likelihood (LK) of each possible combination of genotypes given (6) and the LK of offspring combinations ; (2) ranking the LK values and performing pairwise comparisons with the highest LK using the Akaike Information Criterion (AIC) ; (3) selecting a restricted set of the best combinations with AIC<threshold. At the last generation (most recent common ancestor of the whole population) we determined the best LK value and the resulting most likely matrix of genotypes. An example is given Figure 1. Our motivation to proceed in two steps, retaining first several best combinations at each generation but only a single one at the last generation, was guided by the observation that the most likely combination at a given generation may often not provide the best (most likely) combination at the level of the entire genealogy.

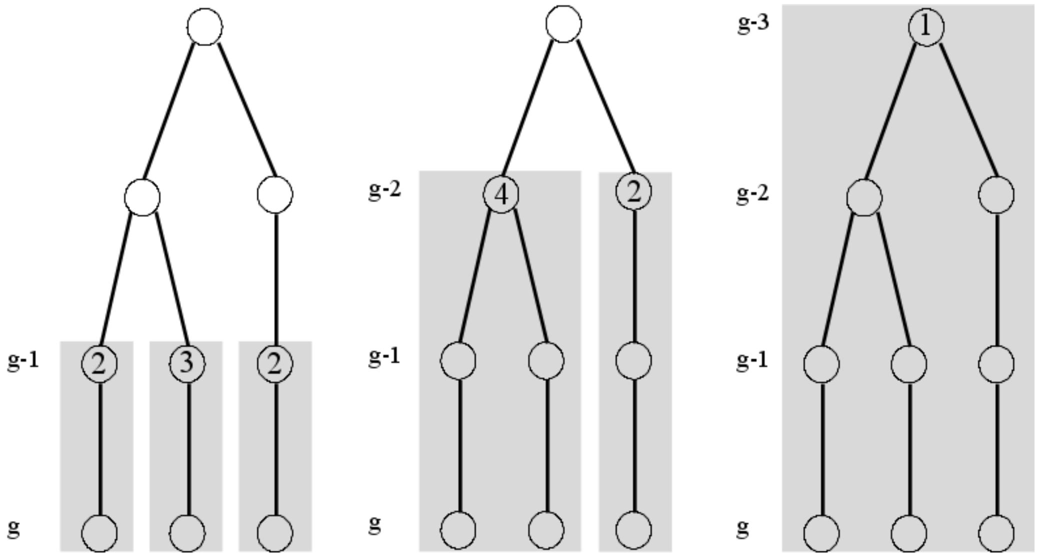

FIGURE 1 – **Principle of the algorithm.** The algorithm functions backward in time. It first considers all possible $(k+1)$-uplets at generation $g-1$ (left panel). Here there are three 2-uplets encompassing a parent at generation g-1 and a single offspring at generation g with 3 possible genotypes (AA, Aa, aa) that is 9 possible combinations for each 2-uplet. After computing LK values for each combinations and keeping only the best ones, we ended-up with respectively 2, 3 and 2 best combinations (AIC<threshold) as indicated in the circles at generation g-1. At generation g-2 (middle panel), there are two $(k+1)$-uplets, a 5-uplet and a 3-uplet. We considered all 3 possible genotypes for the parents at generation g-2 in combination with respectively 6 (2*3) and 2 genotypes for the offsprings at generation g-1 and g, for a total of 18 and 6 combinations of genotypes. Here, we retained respectively 4 and 2 best values (AIC<threshold). Following the same rationale we evaluated 24 combinations of genotypes (3*4*2) at g-3 and kept the best combination on the basis of the LK value (right panel).
